# Effects of insertion torque on the structure of dental implants with different connections: Experimental pilot study in vitro

**DOI:** 10.1371/journal.pone.0251904

**Published:** 2021-05-19

**Authors:** Sergio Alexandre Gehrke, Gismari Miranda Amaral Pereira, Arthur Felipe Gehrke, Nilton De Bortoli Junior, Berenice Anina Dedavid

**Affiliations:** 1 Department of Biotechnology, Catholic University of Murcia, Murcia, Spain; 2 Biotecnos Research Center, Montevideo, Uruguay; 3 Student of Implantology Program, Associação Paulista dos Cirurgiões Dentistas—APCD, São Bernardo do Campo, Brazil; 4 Department of Mathematics, Universidade Federal de Santa Maria, Santa Maria, Brazil; 5 Department of Oral Implantology, Associação Paulista dos Cirurgiões Dentistas—APCD, São Bernardo do Campo, Brazil; 6 Department of Materials Engineering, Pontificial Catholic University of Rio Grande do Sul, Porto Alegre, Brazil; University of Vigo, SPAIN

## Abstract

**Objective:**

During the insertion of dental implants in the bone tissue, different torque values can be applied. However, the high applied torque can cause damage to the implant connection. Our study sought to evaluate, by measuring the angle of rotation of the insertion drive and, later microscopic observation, possible changes in the structure of implants of different diameters with 3 different types of connections after the application of 4 different torque intensities.

**Materials and methods:**

Three hundred tapered dental implants and three hundred insertion drivers were used in the present study. Implants of 3.5 and 4 mm in diameter with 3 connection models were tested: external hexagon (EH), internal hexagon (IH) and Morse taper (MT). Then, sis groups were performed: EH3 group, EH4 group, IH3 group, IH4 group, MT3 group and MT4 group. The samples were submitted to the torque/torsion force at 4 intensities (n = 10 samples per group and intensity): 60, 80, 100 and 120 Ncm. The turning angle of the insertion driver was measured in each test. In addition, in 10 samples from each group, the maximum torque value supported by each implant model was measured. After the tests, all samples were inspected microscopically to describe the observed changes.

**Results:**

The maximum torque supported by the different implant models showed statistically significant difference (p < 0.0001). The values of the measured angles showed statistically significant differences between the torque values applied within each group (p < 0.001) and between groups with the same torque value (p < 0.001).

**Conclusions:**

Within the limitations of the present study in vitro, the results showed that high torque values cause mechanical damage to the implants.

## Introduction

The use of dental implants for the rehabilitation of missing teeth has been a routine in the modern dentistry practice as it is a predictable and long-lasting treatment [1, 2]. However, to obtain implant osseointegration, adequate primary stability plays a critical role and, many clinicians have proposed that greater insertion torque is safer for the osseointegration process to occur properly [3–5]. For the immediate loading technique, the ideal initial stability would be a torque value between 32 and 60 Ncm [6], with some authors reporting that the higher the value of the insertion torque, the greater the success rate [7, 8].

However, identifying the functional and mechanical limitations of implant systems is essential for the long-term success of the restoration [9]. During the insertion of implants into the bone tissue and the application of the torque to obtain the initial stability, the sets (implant and insertion driver) are subjected to torsional forces, which depending on their intensity can affect the structure of these parts [10, 11]. The titanium implants are metallic pieces that have different limited values of resistance to the loads and/or external forces applied on their structure. Obviously, these values vary depending on the design of the implant and its dimensions [9, 12, 13].

Another point that must be considered, is the fact that the implants to be installed in the bone tissue require some driver for transport and insertion, which is connected to the implant to enable these maneuvers. Different driver designs are manufactured to insert the implant, some are connected to an intermediate piece that is attached to the implant, to protect the implant fitting from inadequate forces, and others are drivers that connect directly to the implant. The latter drivers are of multiple use and are manufactured in a metal of high strength and hardness, usually greater than the strength and hardness of the titanium used for the manufacture of the implant.

Then, our study sought to evaluate implants with different connection models and diameters, which present drivers of multiple-use for the implant insertion. For this, different torque values were applied to the implants and the angle of rotation of the wrench was measured, as well as the maximum torque value supported by each implant model tested. Still, descriptive evaluations of the images after the tests were carried out.

## Materials and methods

**Fig 1:**
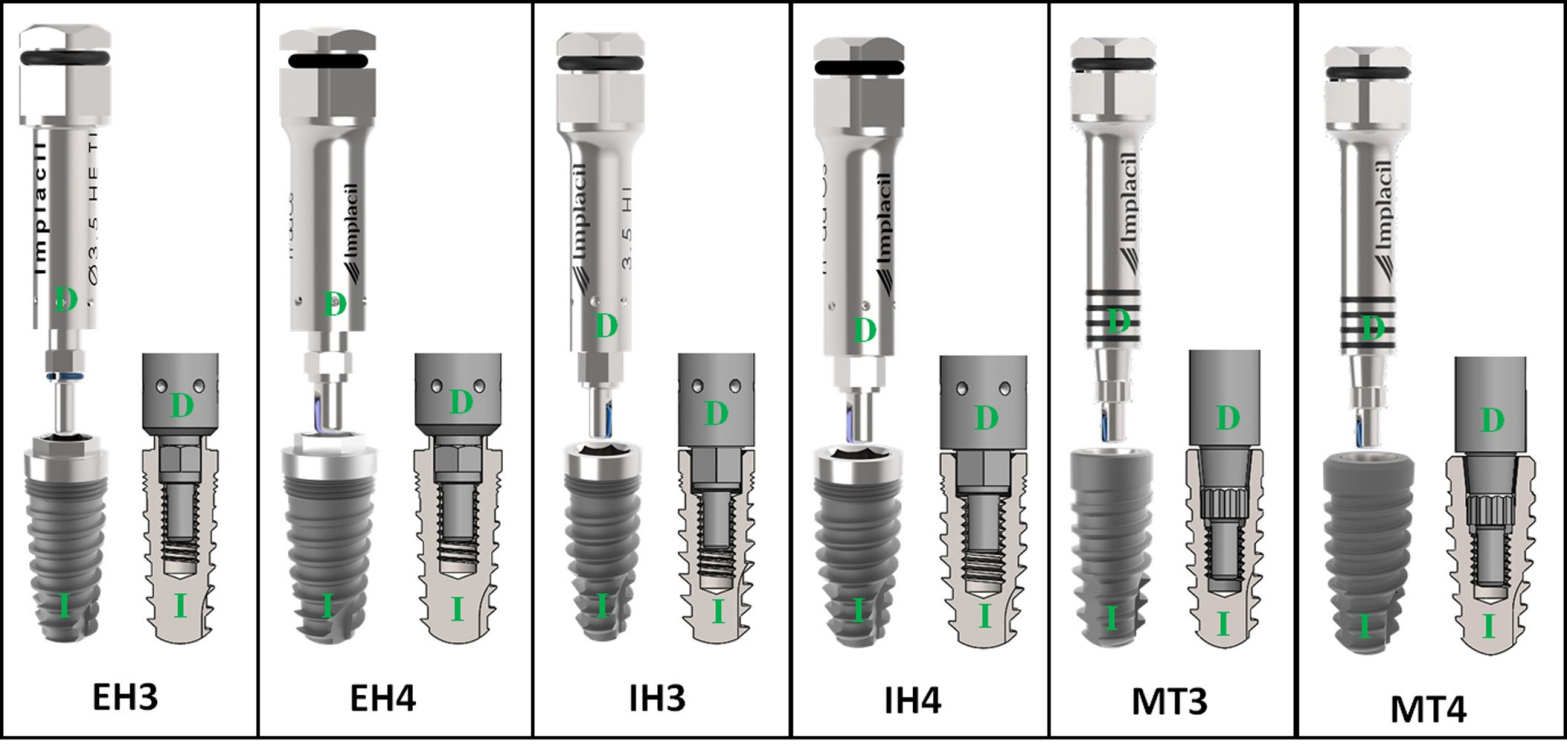
Images of sets and schematic connection of sets (implant and insertion driver) used for the test of each group. Green letters: D = insertion driver and I = implant.

Three hundred dental implants made of grade 4 titanium (ASTM F67) [14] were used in the present study. The Rockwell C hardness of the titanium is 36–38 HRC. All implants used were conical in shape, manufactured by the company Implacil De Bortoli (São Paulo, Brazil). The groups were determined according to the type of connection and the diameter of each sample (n = 11 per group): EH3 group, external hexagon (EH) connection implant with 3.5 mm in diameter; EH4 group, EH implant with 4.0 mm in diameter; IH3 group, internal hexagon (IH) connection implant with 3.5 mm in diameter; IH4 group, IH implant with 4.0 mm in diameter; MT3 group, Morse taper (MT) connection implant with 3.5 mm in diameter; MT4 group, MT implant with 4.0 mm in diameter. For the test, an insertion key was used for each sample, totaling 300 pieces. The used and commercialized insertion drivers were manufactured in wrought stainless steels for surgical instruments ASTM F899 [15], that after receiving the heat treatment it presents a Rockwell C hardness of 49–52 HRC. **Fig 1** shows an image of each implant model tested, its corresponding insertion drivers and a schematic image of the fit between the insertion driver and the implant.

The implants were fixed in a vise of the equipment, being completely static, leaving 3 mm of the cervical portion out. For the test, the ISO/TS 13498:2011 standard was followed [16]. The insertion driver was connected to the implant and fixed to the mechanical clamp for the torque application. Two figures with a schematic image of the positioning of the sets (implant and insertion driver) in the equipment to apply the torque force and measure the angle of rotation and an image of the computerized torque equipment used to perform the tests were added as a **Supplementary Materials**.

**Fig 2:**
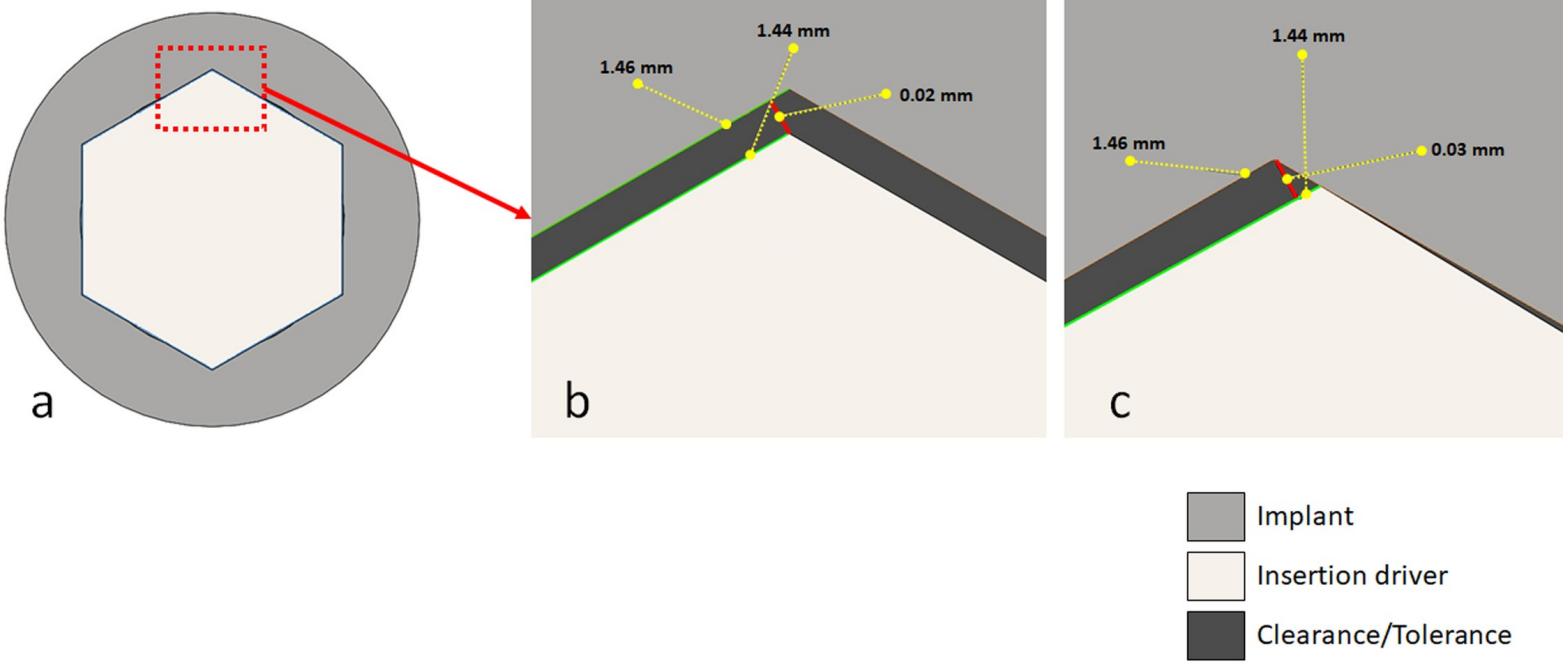
Schematic image of the project design: (a) insertion driver coupled in the implant; (b) space (clearance/tolerance) between the implant and the insertion driver after coupling; (c) the movement generated after the insertion driver rotation to apply the torque.

Five different torque values were predetermined and applied using a Torque Testing Machine–CME (Técnica Industrial Oswaldo Filizola, São Paulo, Brazil). This machine is a fully controlled by the DynaView Torque Standard/Pro M software, which performed the calculations and generated reports automatically. Firstly, the maximum torque (Tmax) that supports each implant model was measured (n = 10 per group). Then, the other samples were submitted to the application of predetermined torque values, which was made with a speed of 1 rpm in 10 samples per group for each value, as follows: torque 1 (T1) at 60 Ncm, torque 2 (T2) at 80 Ncm, torque 3 (T3) at 100 Ncm and, torque 4 (T4) at 120 Ncm. The angle of rotation to reach each torque value was captured for analysis. As there is a space between the diameter of the implant portion to insert the driver (coupling) and the diameter of the driver, called in the mechanical terms as clearance/tolerance, the angle of free rotation was measured for each group (**Fig 2**). For this, after the positioning of the sets on the equipment, an initial torque of 1 Ncm was applied, just to be able to measure this initial rotation angle (tolerance value). Then, the angle and torque meters were reset to start applying the proposed loads.

After applying the corresponding torque value, each sample was placed in a Leica DMS300 digital surface microscope (Leica Microsystems, Wetzlar, Germany) and images were acquired to assess the structural effects of the implants at the insertion driver location.

The sample size was based on a power level of 85% to obtain a *p*-value of 0.05, calculated using the software SigmaStat 4.0 (Systat Software Inc., San Jose, USA). For a desired power level of 85% with differences between the means and standard deviations of each group, the minimum sample size for each group under each condition was 8 samples.

Statistical analysis was performed using the GraphPad Prism software version 5.01 for Windows (GraphPad Software, San Diego, California, USA). Initially, analyses of normality (D’Agostino-Pearson normality test) were performed. Once normality was verified, the parametric generalized linear model for repeated measures was applied at a 5% significance level. The data were compared statistically using the ANOVA One-Way test to verify differences between the 6 groups in the 4 proposed torque values and in maximum torque. Bonferroni multiple comparison test was used to compare the data between the groups. All cases where p <0.05 were considered statistically significant.

## Results

**Fig 3:**
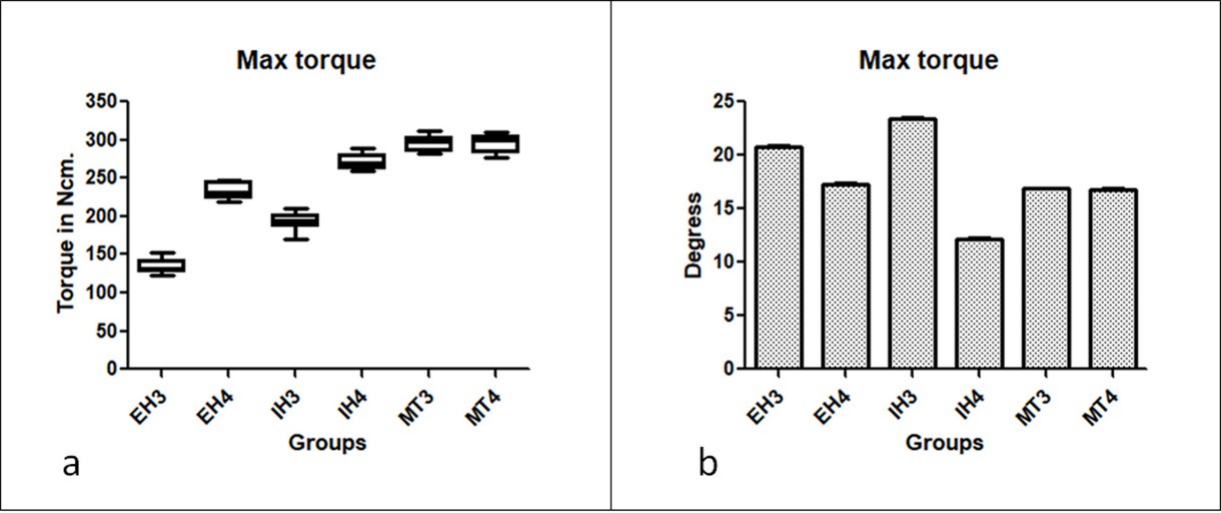
(a) Graph of the data of torque maximum supported in each group; (b) Bar graph of the rotation angle measured until the maximum torque was reached.

In general, the maximum torque values measured for the proposed groups showed a statistically significant difference (p < 0.0001). The MT group implants showed the highest torque values. In the EH and IH groups, the implants with the regular diameter (Ø4.0 mm) presented the highest maximum torque values; while in the MT groups, in both tested diameters, the maximum torque values measured showed very similar values, with no statistical difference between them (p > 0.05). The data of torque maximum supported in each group are summarized in the graph of **Fig 3A**. The table with the exactly calculated values is presented as a **Supplementary Materials**.

**Fig 4:**
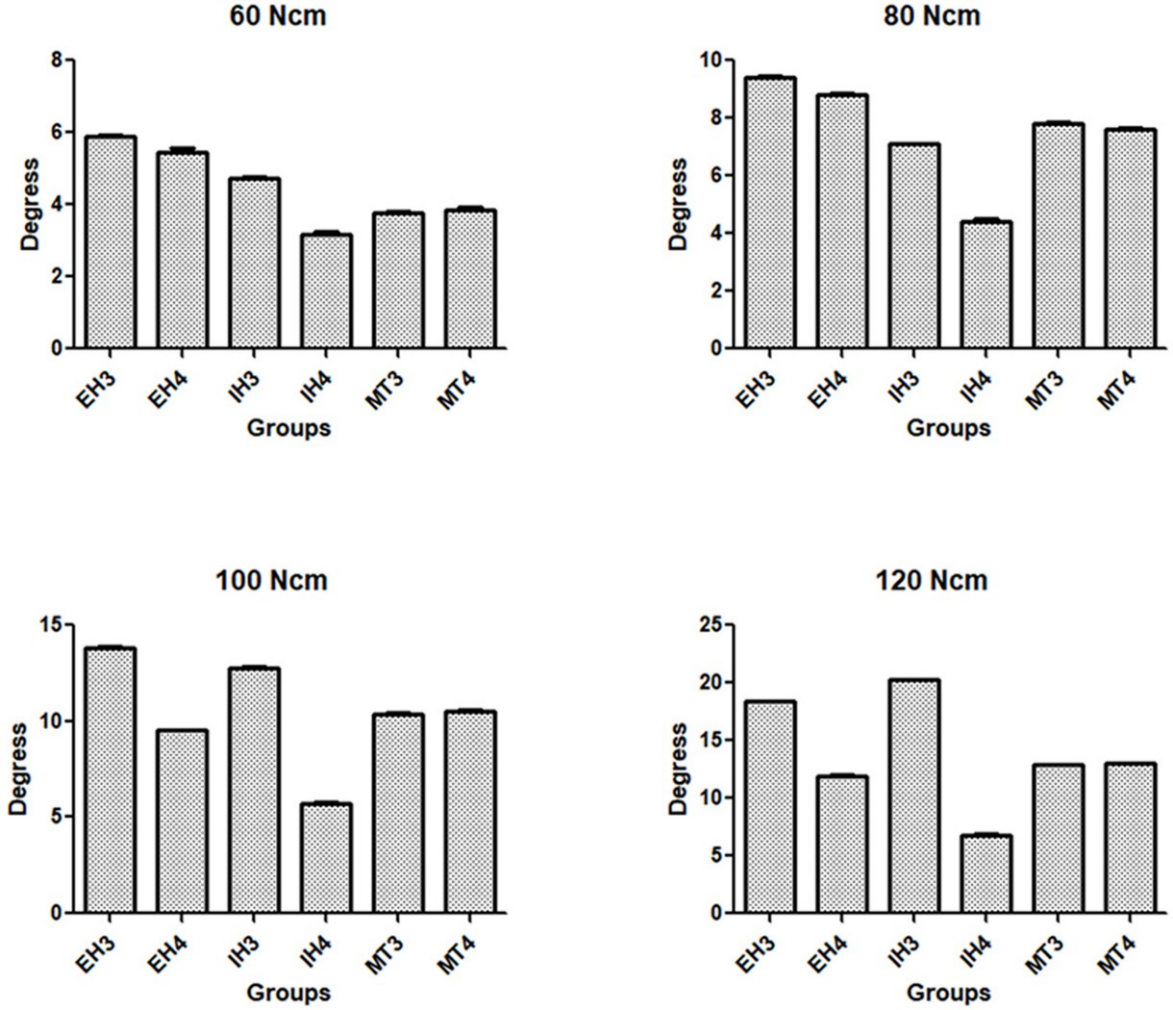
Bar graphs of the mean and standard deviation data of the measured rotation angle in each applied torque value.

The calculated mean and standard deviation of the angle measured applied the initial torque of 1 Ncm, corresponding to the clearance/tolerance between all sets (implant and driver), was of 3.62 ± 0.08 degrees. **Fig 3B** show the rotation angle measured until the maximum torque was reached. The values of the measured angles showed statistically significant differences between the torque values applied within each group (p < 0.001) and between groups with the same applied torque value (p < 0.001). However, when comparing only the groups with the same connection model using the Bonferroni multiple comparison test, only the MT groups showed values without statistical differences in all applied torque levels (p > 0.05). **Fig 4** show graphically the mean and standard deviation data of the measured rotation angle for each applied torque value. The table with the exactly calculated values is presented as a **Supplementary Materials**.

**Fig 5:**
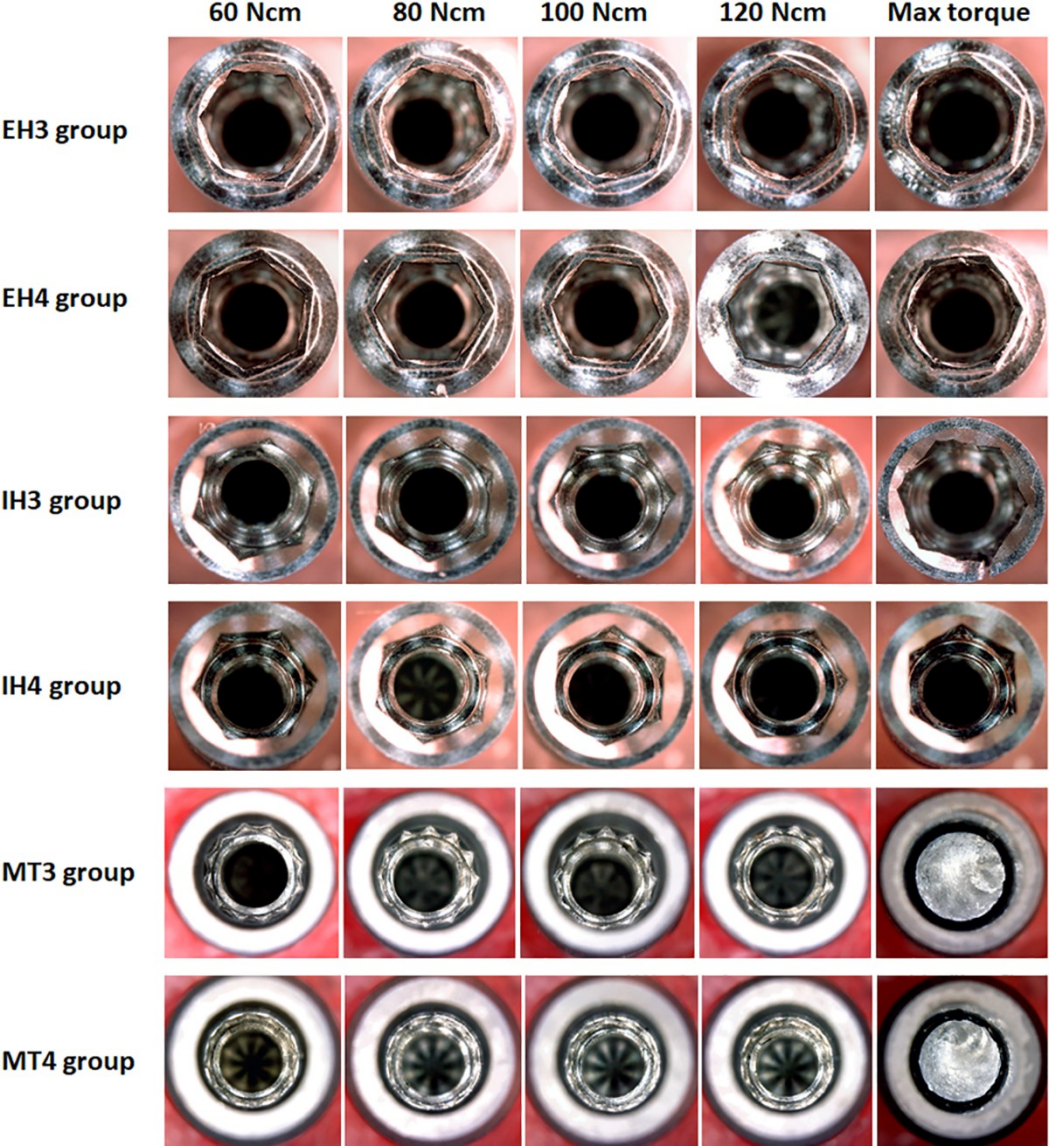
Representative images of the samples of each group are shown after the application of the predetermined torque values.

**Fig 5** show representative images of the samples of each group are shown after the application of the predetermined torque.

**Fig 6:**
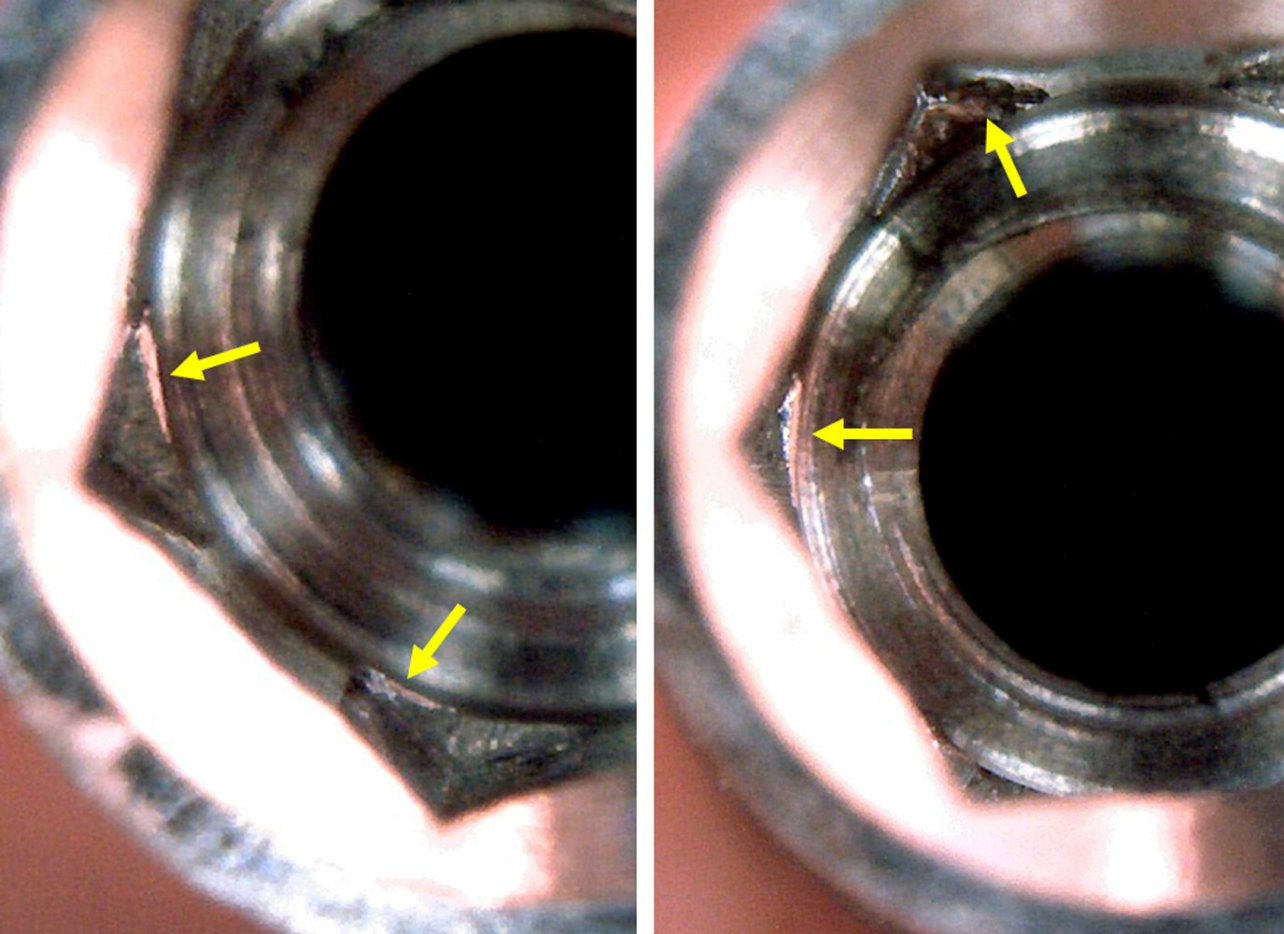
Representative image of samples after the application of 60 and 80 Ncm of torque, respectively. Yellow arrows show the marks in the local where the insertion drive made the force.

In all samples of all groups the application of torque with 60 and 80 Ncm, only marks were observed on the walls where the insertion driver made contact with the implant, without causing major changes, as shown in **Fig 6**.

**Fig 7:**
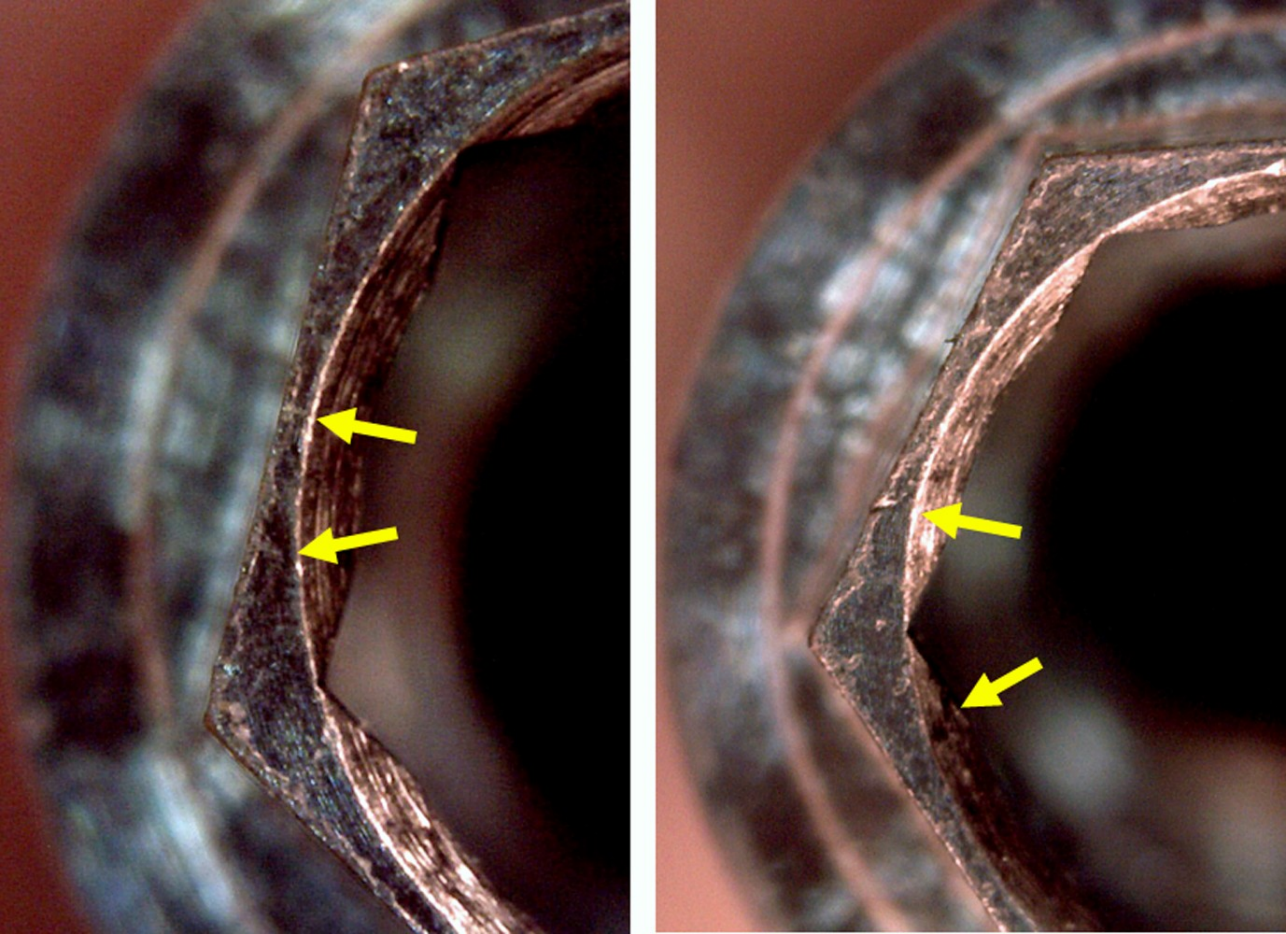
Representative image showing what happened in the samples of groups EH and IH after the application of 100 and 120 Ncm of torque, respectively. Yellow arrows show the marks in the local where occurred the fissures in the implant hexagon.

**Fig 8:**
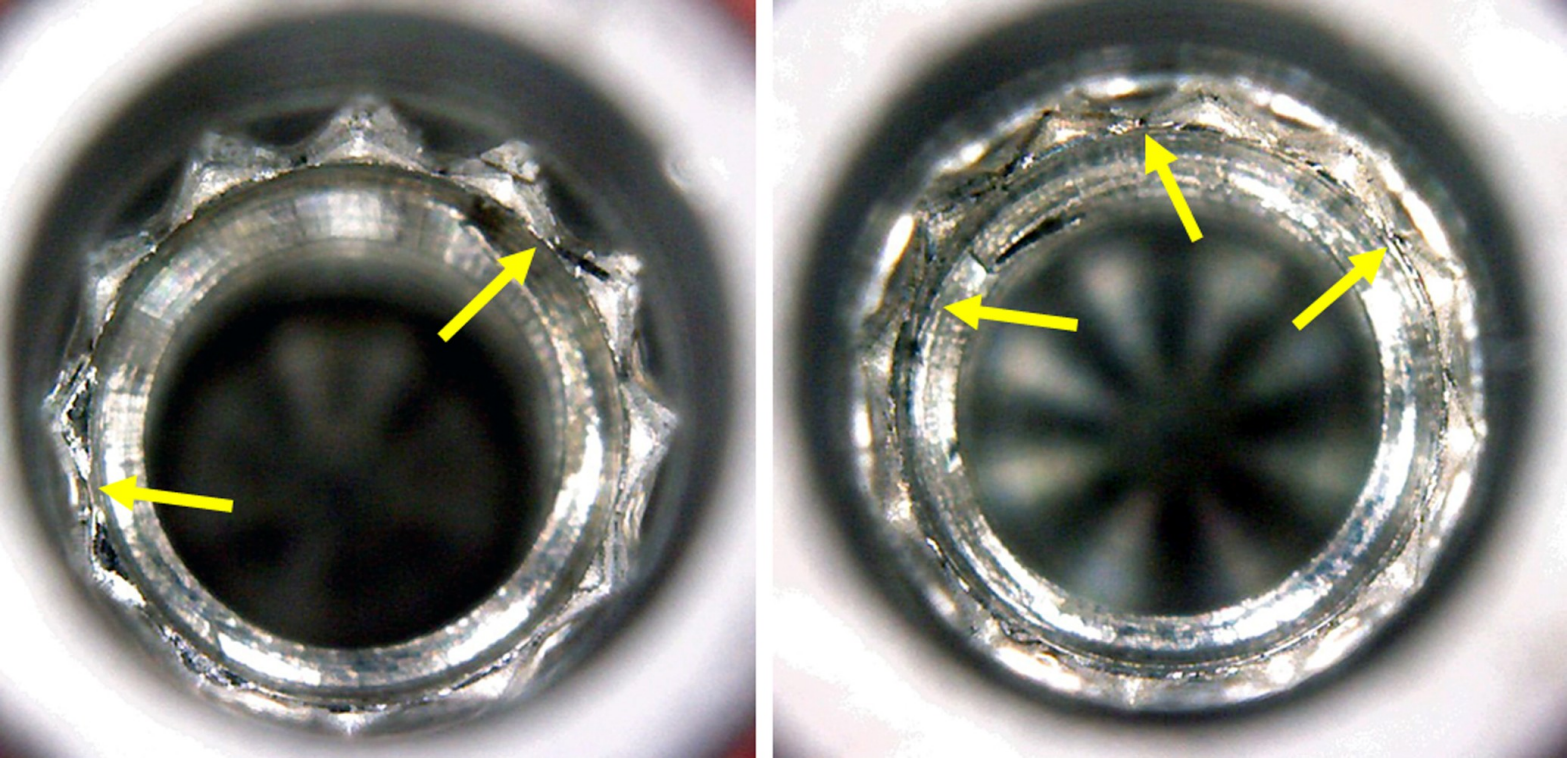
Representative image showing the rounding of angles (yellow arrows) in the samples of both MT groups after the application of 100 and 120 Ncm of torque, respectively.

In the application of torque with 100 and 120 Ncm, all samples showed rounding of the angles where the insertion driver made the force on the implant. In the narrow diameter implants (Ø 3.5 mm) of the EH and IH groups, in addition to rounding, cracks were observed in the implant hexagons (**Fig 7**). While in the samples of both MT groups, the dodecagon where the driver was inserted showed rounding of its angles (**Fig 8**).

**Fig 9:**
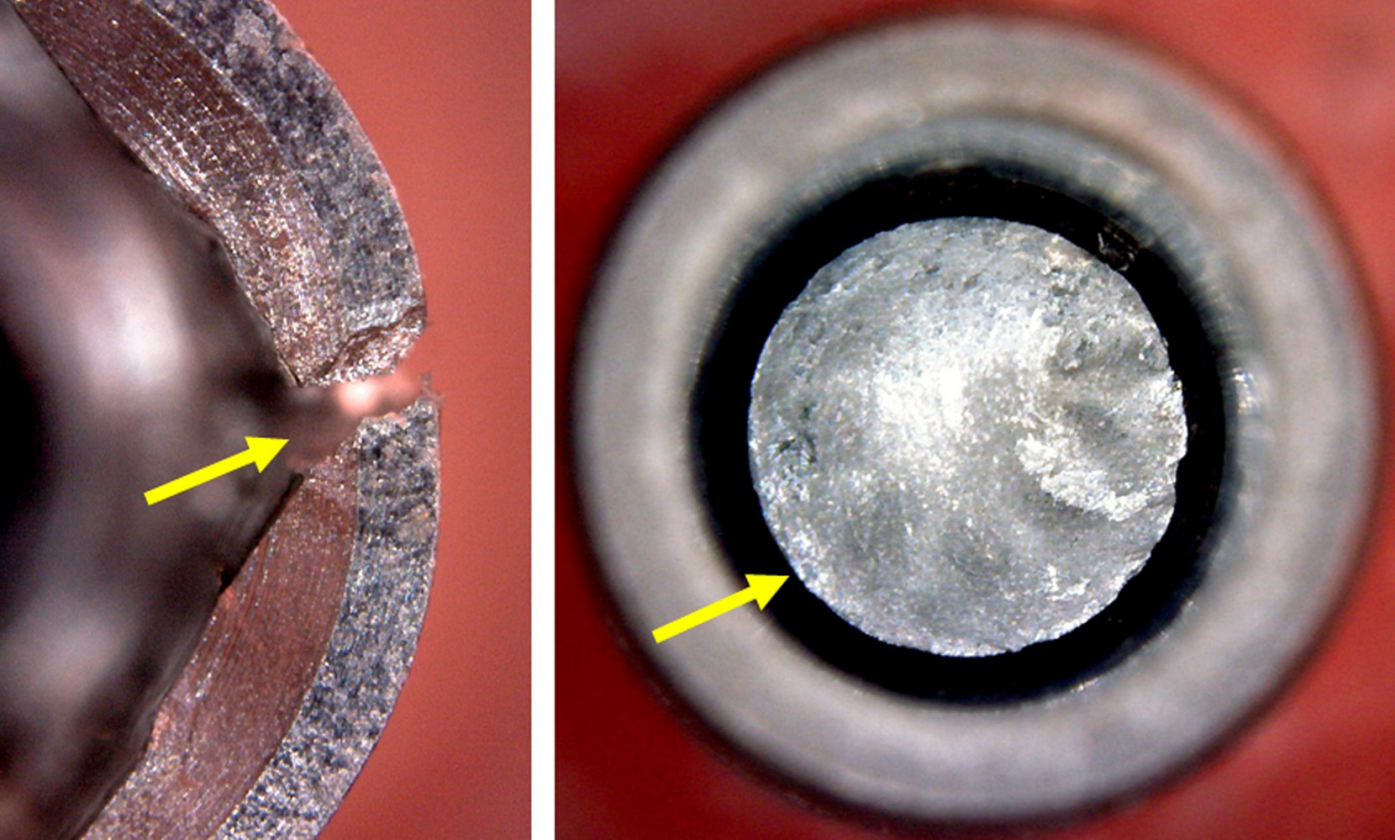
Representative images of the maximum torque applied in the implants: (a) image of an implant sample with hexagonal connection (IH3 group) showing a complete fracture of the implant wall (yellow arrow); (b) image of a sample of Morse taper connection (MT3 group) showing the fracture of the insertion driver inside the implant (yellow arrow).

While in the samples that received the maximum torque, the implants of the EH and IH groups showed fractures in the hexagon and, in the implants of both MT groups, the insertion drivers have broken (**Fig 9**).

## Discussion

This experimental study aimed to evaluate implants with three different connection models in two diameters (3.5 and 4.0 mm), which present insertion drivers of multiple-use for the implant insertion. Different torque values were applied to the implants and the rotation angle of the wrench was measured, as well as the maximum torque value supported by each implant model tested. The magnitude of the deformations, and the characteristics of the deformations were evaluated by photographic images. The results showed that, regardless of the type of implant connection, insertion torque values above 80 Ncm affect the implant structure, corroborating findings published in other studies [11]. These changes caused in the implants can lead to clinical problems, such as loosening of abutments and/crowns, fracture of abutment fixation screws, problems with the adaptation of abutments, bacterial infiltration between the sets, among others [17, 18].

The precision of the sets (implant and abutment) is fundamental for the long-term success of the rehabilitation treatment with implants [19]. Deformations and/or changes in the connection system of the implants with the abutment can affect the results and the longevity of rehabilitation treatments with dental implants. In addition to the possible mechanical failures caused by the mismatch of the IA sets [17], biological problems can arise, such as possible peri-implant changes due to the passage of bacteria and their fluids [20, 21]. On the other hand, the insertion drivers were not inspected in this study because their changes do not interfere with the results and treatment longevity.

Regarding the maximum supported torque, the results showed higher torque values supported by the MT group implants are surely explained by the location where the driver is inserted, that is, inside the implant where the implant thickness is greater, decreasing the possibility of cracks or fractures in the implant. While in groups EH and IH, the local where the insertion drivers are positioned is in the most fragile portion of the implant. These results corroborate the findings published in other studies [11], which demonstrated that the Morse taper implant connection showed the lowest deformation rates in comparison with the internal and external hexagon implants.

The degree of rotational freedom between the implant and the prosthetic abutment is considered as an important factor determining the long-term stability between the two parts (implant and abutment) and, the increased rotational freedom has been associated with higher rate of screw loosening [17]. Regarding the rotation angle, our results showed that almost all groups (except the IH4 group) presented the values of the rotation angle at maximum torque applied very close to the values obtained with the application of torque of 120 Ncm, showing that this value torque is not supported and should not be applied to these implant models. Our results obtained for the IH4 group, with lower rotation values and, consequently, less damage to the hexagon angles, were similar to the results published by other authors who evaluated implants with the same type of connection and diameter [22]. Moreover, in all groups proposed, the value of the rotation angle measured during the application of the proposed torque values was low in the 60 and 80 Ncm of loads, greatly increasing the values in the 100 and 120 Ncm of loads.

Some studies have shown that high torque values do not affect the osseointegration of the implants [23–25], including being able to improve the bone healing process by the micro-fractures generated in the bone tissue [25]. These studies had a wide range of IT (>25–176 Ncm), and there was no consensus on what was considered a high insertion torque value [24–27]. However, with the results obtained in our study, we can determine that, mechanically, values above 80 Ncm should be considered as high torque values, as the implants have undergone changes that are likely to affect the long-term performance of these parts. In this sense, other authors showed that a geometric deformation of the anti-rotational system can compromise its function after a torque magnitude of 36 Ncm [28]. Finally, the clinician must be aware of the force limits supported by the implants if he wants to install the implants with high torque values, as the different models can present deformations in the fitting system of the prosthetic abutments and precision problems. As described by Kourtis and Collaborators [17], the inaccuracy of fit in the implant-abutment interface can increase the degree of rotational freedom and the risk for long-term success of the restoration.

Among the limitations of the present in vitro study, we can describe that the torque application was performed on the implant completely static, which in a clinical situation does not occur. However, new studies could be carried out to verify whether the fact that the implant is in motion during insertion, added to the elasticity of bone tissue, could absorb some of this stress from the applied load. Long-term clinical studies evaluating the stability of abutments and rehabilitation in cases of implants installed with high torque should be proposed.

## Conclusions

Within the limitations of the present study in vitro, the results showed that high torque values cause mechanical damage to the implants. The implants of external and internal hexagon connections, mainly of reduced diameter (3.5 mm), present important deformations in the hexagon when torque forces above 80 Ncm are applied. The application of loads with values of 100 and 120 Ncm caused rounding of the fitting systems and cracks in all implants models tested.

## Supporting information

S1 Fig Schematic image of the positioning of the sets (implant and installation driver) in the equipment to apply the torque force and measure the angle of rotation. (DOCX)

S2 Fig Image of the computerized torque equipment used to perform the tests. (DOCX)

S1 Table S1 Table of the Fig 3A. Mean, standard deviation (SD) and median obtained of maximum torque supported in each group (values in Ncm).
(DOCX)

S2 Table S2 Table of the Fig 4. Mean, standard deviation and median of the rotation angle in degrees measured in each group with different torque levels proposed.
(DOCX)
