# Supplementary material for: Effects of insertion torque on the structure of dental implants with different connections: Experimental pilot study in vitro
Source: PLoS One. 2021 May 19;16(5):e0251904. doi: 10.1371/journal.pone.0251904 (PMC8133438; doi:10.1371/journal.pone.0251904)
Supplement: S1 Fig — (DOCX) [file pone.0251904.s001.docx]

**
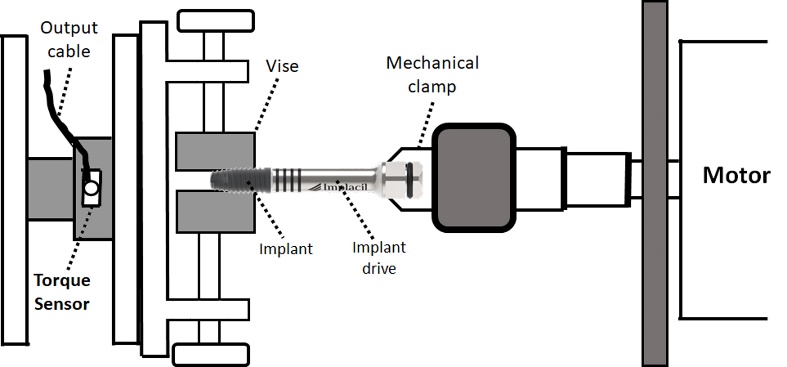
**

**S1 Fig.** Schematic image of the positioning of the sets (implant and installation driver) in the equipment to apply the torque force and measure the angle of rotation.
