# Supplementary figures and images for: Effects of insertion torque on the structure of dental implants with different connections: Experimental pilot study in vitro
Source: PLoS One. 2021 May 19;16(5):e0251904. doi: 10.1371/journal.pone.0251904 (PMC8133438; doi:10.1371/journal.pone.0251904)

**
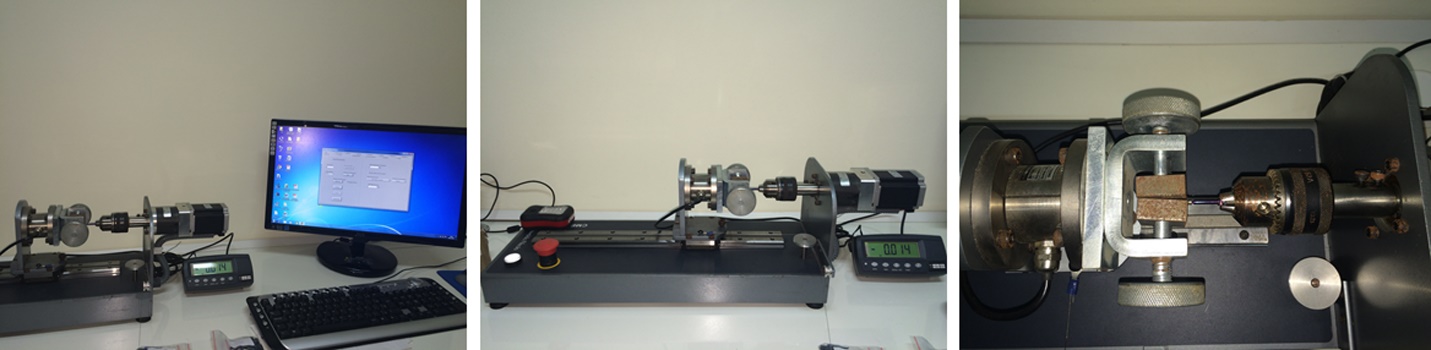
**

**S2 Fig.** Image of the computerized torque equipment used to perform the tests.

Supplement: S2 Fig — (DOCX) [file pone.0251904.s002.docx]
