# Supplementary material for: Effects of insertion torque on the structure of dental implants with different connections: Experimental pilot study in vitro
Source: PLoS One. 2021 May 19;16(5):e0251904. doi: 10.1371/journal.pone.0251904 (PMC8133438; doi:10.1371/journal.pone.0251904)
Supplement: S1 Table — Mean, standard deviation (SD) and median obtained of maximum torque supported in each group (values in Ncm). (DOCX) [file pone.0251904.s003.docx]

**S1 Table. Supplementary Table 1 of the Figure 3a.** Mean, standard deviation (SD) and median obtained of maximum torque supported in each group (values in Ncm).

| **Group** | **EH3** | **EH4** | **IH3** | **IH4** | **MT3** | **MT4** |
| --- | --- | --- | --- | --- | --- | --- |
| **Mean** | 134.3 | 233.4 | 193.0 | 271.8 | 296.4 | 295.5 |
| **SD** | 9.2 | 10.7 | 10.9 | 9.2 | 10.3 | 11.0 |
| **Median** | 132.2 | 231.7 | 194.0 | 270.0 | 297.0 | 299.2 |
