# Supplementary material for: Effects of insertion torque on the structure of dental implants with different connections: Experimental pilot study in vitro
Source: PLoS One. 2021 May 19;16(5):e0251904. doi: 10.1371/journal.pone.0251904 (PMC8133438; doi:10.1371/journal.pone.0251904)
Supplement: S2 Table — Mean, standard deviation and median of the rotation angle in degrees measured in each group with different torque levels proposed. (DOCX) [file pone.0251904.s004.docx]

**S2 Table. Supplementary Table 2 of the Figure 4.** Mean, standard deviation and median of the rotation angle in degrees measured in each group with different torque levels proposed.

| **Group** | **60 Ncm** | **80 Ncm** | **100 Ncm** | **120 Ncm** | **Max torque** |
| --- | --- | --- | --- | --- | --- |
| **EH3** | 5.9 ± 0.1 (5.9) | 9.4 ± 0.2 (9.4) | 13.8 ± 0.4 (13.8) | 18.3 ± 0.2 (18.3) | 20.7 ± 0.4 (20.6) |
| **EH4** | 5.5 ± 0.4 (5.4) | 8.8 ± 0.2 (8.9) | 9.5 ± 0.2 (9.5) | 11.9 ± 0.2 (11.9) | 17.3 ± 0.3 (17.7) |
| **IH3** | 4.7 ± 0.2 (4.8) | 7.1 ± 0.1 (7.1) | 12.7 ± 0.3 (12.7) | 20.2 ± 0.2 (20.3) | 23.4 ± 0.4 (23.4) |
| **IH4** | 3.2 ± 0.3 (3.3) | 4.4 ± 0.3 (4.5) | 5.7 ± 0.3 (5.7) | 6.2 ± 0.4 (6.8) | 12.1 ± 0.2 (12.0) |
| **MT3** | 3.7 ± 0.2 (3.7) | 7.8 ± 0.2 (7.9) | 10.3 ± 0.3 (10.3) | 12.9 ± 0.2 (12.9) | 16.9 ± 0.3 (16.8) |
| **MT4** | 3.9 ± 0.2 (3.9) | 7.6 ± 0.2 (7.6) | 10.5 ± 0.2 (10.5) | 13.0 ± 0.2 (13.0) | 16.8 ± 0.3 (16.8) |
